# The plastisphere and river systems as reservoirs for antibiotic resistant bacteria

**DOI:** 10.3389/fmicb.2025.1721325

**Published:** 2026-01-22

**Authors:** Soraya Alfonsi, Francesca Racciatti, Frank Guzman, Attilio Fabbretti, Pohl Milon, Luca Agostino Vitali, Roberto Spurio, Dezemona Petrelli

**Affiliations:** 1 School of Biosciences and Veterinary Medicine, University of Camerino, Camerino, Italy; 2 Biomolecules Laboratory, Faculty of Health Sciences, School of Biology, Universidad Peruana de Ciencias Aplicadas, Lima, Peru; 3 School of Pharmacy, University of Camerino, Camerino, Italy

**Keywords:** 3GC-cephalosporins, plastic pollution, antimicrobial resistance, environmental biofilm, Class 1 Integron, riverine ecosystem, resistome, enterobacteriaceae

## Abstract

Antimicrobial resistance (AMR) is a critical global health threat. This phenomenon involves the diffusion of bacteria and genes among humans, animals and the environment. In particular, the presence of third generation cephalosporin (3GC)-resistant *Enterobacteriaceae* in natural environments is of high concern as they are classified as critical-priority pathogens of public health importance. In this work we studied the relation among plastic pollution in freshwater ecosystems, the spread of multidrug-resistant (MDR) bacteria and diffusion of antibiotic resistance genes (ARGs). Caged plastic fragments were deliberately introduced in a river of central Italy. Plastic samples were collected and analyzed in parallel with river water samples. Out of 267 cefotaxime (CTX) resistant isolates obtained, 65 CTX-resistant *Enterobacteriaceae* were selected for further analysis. Most of the isolates (75% of plastic-derived and 84% of water-derived isolates) were MDR with seven being carbapenem-resistant enterobacteria (CRE). Five of them synthesize KPC (*Klebsiella pneumoniae* carbapenemases) enzymes, and two strains were positive for metallo-β-lactamases (NDM). Among the KPC producers, three isolates were identified as *K. pneumoniae* sequence type ST1519. Their isolation in a natural ecosystem is alarming because they can potentially re-enter human populations through environmental pathways. Shotgun metagenomic analysis provided a comprehensive snapshot of the microbial communities associated to the plastisphere, revealing dominance of families such as *Comamonadaceae*, *Sphaerotilaceae*, and *Flavobacteriaceae*, which play key roles in environmental biofilm formation and stability. The resistome analysis highlighted the presence of ARGs conferring resistance to clinically important antibiotics, such as beta-lactams, vancomycin, and tetracyclines, alongside mobile genetic elements (MGEs) such as integrons, which facilitate the horizontal transfer of resistance genes. This study provides crucial experimental evidence that riverine plastic debris acts as a genetic reservoir and could act as an efficient vehicle for the accumulation and transfer of clinically relevant resistance determinants.

## 1 Introduction

Antimicrobial resistance (AMR) has become one of the most critical public health threats of the 21st century, requiring immediate global action as highlighted by the World Health Organization (WHO) and the European Centre for Disease Prevention and Control (ECDC) (European Centre for Disease Prevention and Control [ECDC], 2023; World Health Organization [WHO], 2024). Its rise is associated with high treatment failures and recurring infections, higher morbidity and mortality rates as well as with increasing healthcare costs (Huemer et al., 2020; Ding et al., 2023). It is estimated that by 2050, untreatable AMR infections will directly cause a 1.9 million deaths and be associated with an additional 8.2 million deaths globally each year (GBD Antimicrobial Resistance Collaborators, 2021).

This crisis extends beyond clinical settings with the environment significantly contributing to the emergence and spread of multidrug-resistant (MDR) bacteria and antibiotic resistance genes (ARGs) (Zarean et al., 2025). Therefore, addressing AMR in a One Health perspective is essential to promote a comprehensive strategy that links human, animal, and environmental settings (Finley et al., 2013). In particular, the environmental resistome may represent a rich source for the acquisition of antibiotic resistance determinants by clinically relevant bacterial strains found in natural environments (Larsson and Flach, 2022; Nappier et al., 2020). Freshwater systems collecting pollutants from wastewater treatment plant effluents, healthcare facilities, industrial processes, and agriculture activities, represent a significant route through which resistant bacteria can spread (Andersson and Hughes, 2012). These pollutants (e.g., antibiotic residues, heavy metals, and pesticides) exert selective pressure on microbial communities and drive the co-selection of resistant determinants (Zarean et al., 2025; Zainab et al., 2020).

A factor amplifying the AMR expansion is the biofilm formation. These assemblages of microorganisms, characterized by the proximity of cells, extracellular DNA (eDNA) and heterogeneous extracellular polymeric substances matrix (EPS), promote the ideal conditions for genetic exchange and spread of resistance genes (Flemming et al., 2021). Additionally, EPS creates a physical barrier that can neutralize antimicrobial agents or limit their diffusion, increasing the bacterial tolerance to antibiotics and favoring the persistence of resistance strains (Zhao et al., 2023). In the environment, these communities grow on various surfaces, including biotic and abiotic structures like rocks and artificial substrates such as plastic debris floating in aquatic systems (Besemer, 2015; Michels et al., 2018; Oberbeckmann et al., 2014). Plastic materials, due to their strong durability and physical properties as hydrophobicity allow bacteria to attach and develop. Moreover, the ability to adsorb organic material and pollutants, provides a suitable nutrient source for biofilm growth (Sooriyakumar et al., 2022). Specifically, biofilms growing on plastic, known as *plastisphere*, create a microenvironment where genetic exchange of ARGs via horizontal gene transfer (HGT) is promoted (Amaral-Zettler et al., 2020; Zettler et al., 2013).

HGT is amplified by mobile genetic elements (MGEs) as transposons, integron gene cassette arrays, and plasmids (Partridge et al., 2018). Class 1 Integrons, through site-specific recombination events within their gene cassette, play a central role in the acquisition and expression of ARGs (Bhat et al., 2023). This genetic structure is the most prevalent in MDR clinical isolates and the *intI1* gene, encoding for the enzyme integrase, may be considered as a promising indicator of the anthropogenic pollution levels (Gillings et al., 2015).

**FIGURE 1:**
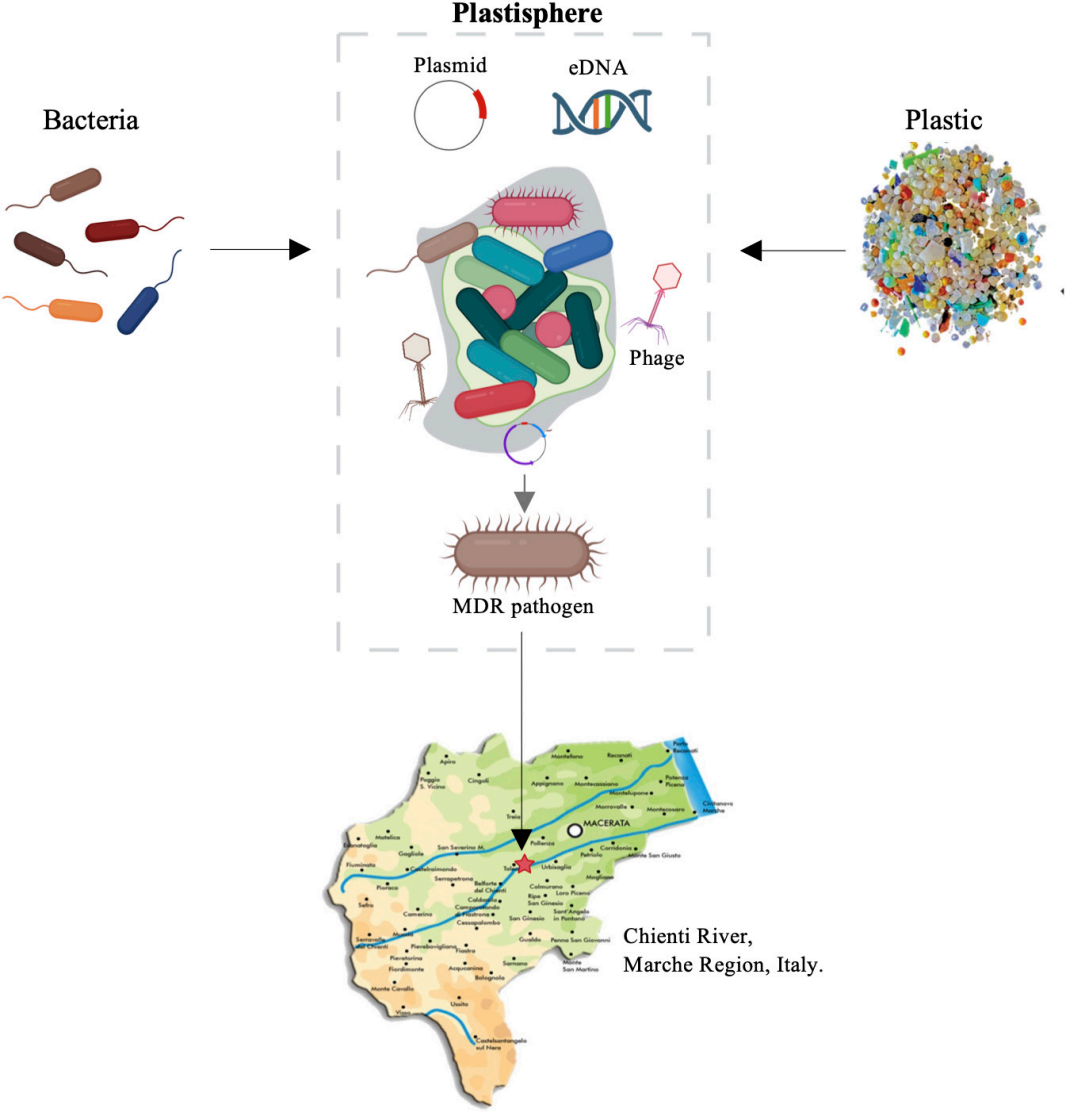
The plastisphere and AMR spread. Schematic diagram showing factors influencing the AMR spread in river ecosystems caused by the formation of biofilm on plastic debris (plastisphere) and horizontal transfer of Antimicrobial Resistance Genes (ARGs) by Mobile Genetic Elements (MGEs). Plastic fragments, deliberately placed into the Chienti river in the Marche region (Italy), and their associated plastisphere were analyzed by a multidisciplinary approach.

The interplay of all the described factors may create a dynamic system where rivers are central conduits for both pollutants and resistant bacteria. Plastic materials introduced in freshwater environments, constitute a substrate for the whole cycle of biofilm development, which in turns may facilitates the HGT of antibiotic-resistance genes by MGEs (Figure 1). Additionally, due to their physicochemical characteristics, plastic particles float and move through the rivers, interacting with the surrounding microbial communities and enhancing the spread of AMR across various environments.

According to the WHO’s classification of critical-priority bacteria, which includes third generation cephalosporin (3GC)-resistant *Enterobacteriaceae* (Tacconelli et al., 2018), we focused our research on the characterization of these resistant bacteria isolated from plastics submerged in a river of central Italy. To this purpose, caged plastic fragments were introduced into different sites along the Chienti river (Marche, Italy). At different time points plastics were collected, and the associated bacteria have been analyzed. In parallel, bacteria from river water samples were recovered and characterized. Our research aimed to: (i) investigate the presence and distribution of 3GC-resistant *Enterobacteriaceae*, (ii) characterize the antibiotic resistance profile of 3GC-resistant isolates at phenotypic and genotypic level, (iii) explore the molecular elements potentially involved in the spread of resistance determinants, and (iv) use metagenomic approaches to provide a comprehensive analysis of the plastisphere, investigating the bacterial communities and the resistome associated to these efficient vehicles for ARGs transfer.

This multidisciplinary approach aims to address knowledge gap providing additional evidence of the spread of clinically significant resistance determinants mediated by rivers and floating plastic debris.

## 2 Results

### 2.1 Distribution and organization of biofilms on plastics

The presence of viable bacteria on plastic surfaces for each sampling was assessed using CLSM (Confocal Laser Scanning Microscopy). This analysis confirmed microbial colonization of both polyethylene (PE) and polypropylene (PP) fragments. Biofilm adhering to plastic substrates were detected in each image with differences in the spatial distribution of the cells and dimension of the clusters observed between the plastic fragments. Specifically, PP showed localized clusters of cells, while PE demonstrated a more homogeneous distribution of the cells on its surface (Supplementary Figure 1).

### 2.2 Isolation and identification of 3GC-resistant *Enterobacteriaceae*

The presence of 3GC-resistant enterobacteria in both the plastic associated biofilms and in the river water was assessed by using chromogenic selective media supplemented with cefotaxime. Out of 267 CTX-resistant isolates collected from both plastic fragments and river water, 65 strains identified as 3GC-resistant *Enterobacteriaceae* were selected for phenotypic analysis: 21 from PP, 19 from PE and 25 from river water. The biochemical identification showed the presence of *Escherichia coli* (18), *Klebsiella pneumoniae* (11), *Enterobacter aerogenes* (6), *Citrobacter freundii* (3), *Citrobacter brakii* (1), *Enterobacter cloacae* (1) associated to plastic, while *Escherichia coli* (16)*, Klebsiella pneumoniae* (7), *Enterobacter cloacae* (1), and *Citrobacter freundii* (1) were detected in river water.

### 2.3 Antimicrobial resistance profiles of 3GC-resistant *Enterobacteriaceae*

**FIGURE 2:**
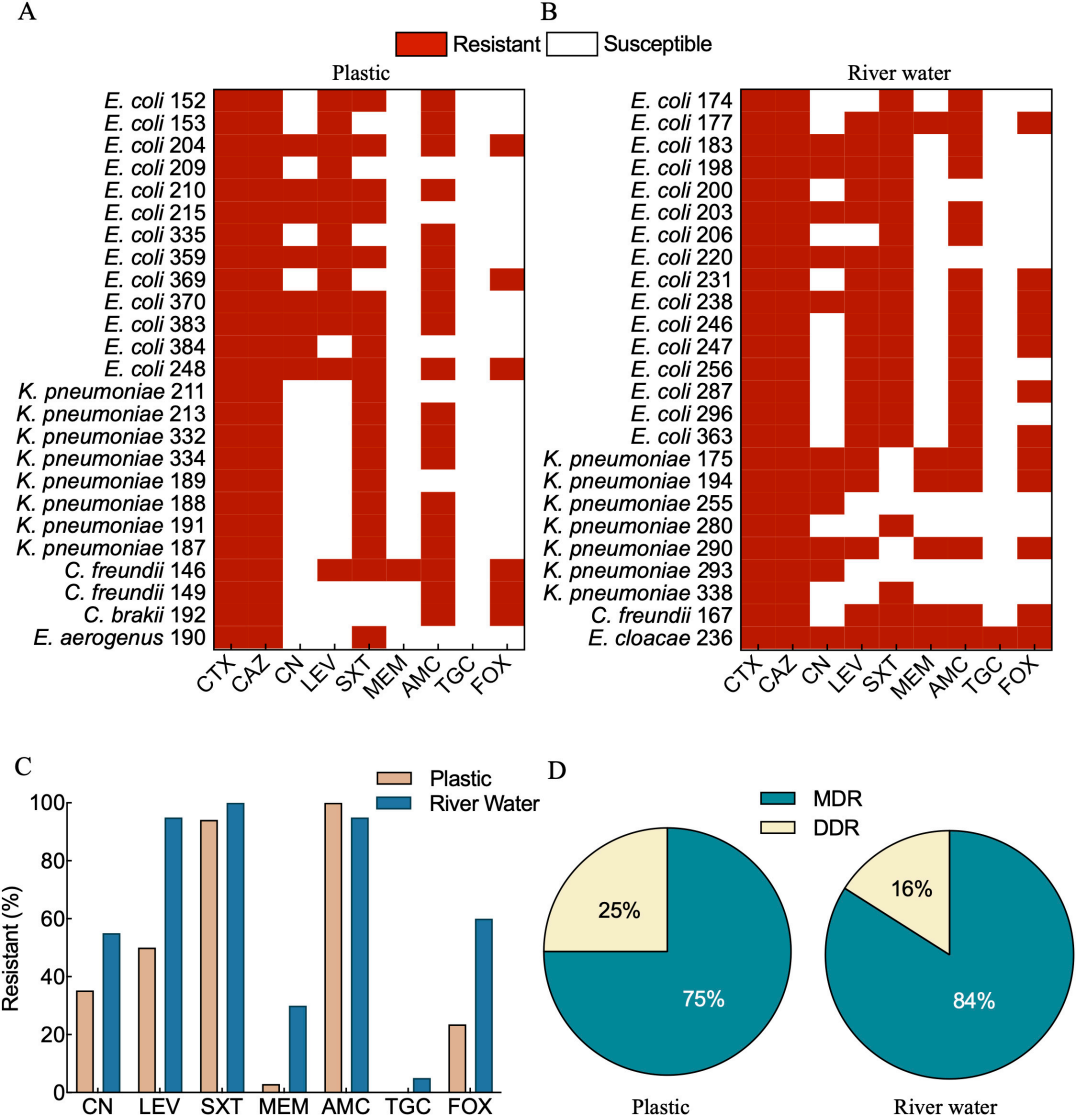
Characterization of 3GC-Resistant *Enterobacteriaceae* isolated from plastic and river water samples. AST performed according to EUCAST guidelines on bacterial strains isolated from plastic **(A)** and river water **(B)**. All the 3GC-resistant *Enterobacteriaceae* were analyzed by disk diffusion test against eight antibiotics covering six drug classes (cephalosporin, aminoglycoside, fluroquinolone, sulfonamide, carbapenem, penicillin and tetracycline). For each group, 25 representative isolates were analyzed. Resistance rates of antibiotics other than cefotaxime and ceftazidime **(C)**. Drug resistance classification types of isolates: Multi-Drug Resistant (MDR) and Dual-Drug Resistant (DDR) **(D)**.

The biochemically identified isolates, were tested for AST to assess their resistance profiles. The results indicated the presence of a high number of multiple resistances associated to isolates from both sample sources, plastic and river water (Figures 2A,B). Antibiotics with the higher resistant rates, other than cefotaxime, were Trimethoprim/sulfamethoxazole (26%), Amoxicillin/clavulanic acid (26.5%), and levofloxacin (18%). Notably, 7 strains (1 from plastics and 6 from river water) were resistant to meropenem, a last resort antibiotic for the treatment of multi-drug-resistant infections (Papp-Wallace et al., 2011). Among the two sources (plastics and river water), trimethoprim-sulfamethoxazole (SXT) showed similar resistance rates. However, amoxicillin (AMC) resistance was detected with higher prevalence in plastic associated isolates, while *Enterobacteriaceae* isolated from river water showed higher resistance rates to cefoxitin (FOX), meropenem (MEM), levofloxacin (LEV), tigecycline (TGC) and gentamicin (CN) (Figure 2C). A significant percentage of 3GC-resistant isolates exhibit MDR traits (72 and 84% for plastic and isolates from river water, respectively), while a minority display dual-drug resistance (DDR) (28 and 16% for isolate from plastic and river water, respectively) (Figure 2D). Slightly different resistance rates were observed between polymer types, specifically PE isolates showed higher resistance to levofloxacin (68%) compared to PP isolates (19%), whereas trimethoprim-sulfamethoxazole (SXT) resistance was more frequent in PP (85.7%) than in PE (73.7%). Cefoxitin resistance was higher in PP-associated isolates (28.6%) compared to PE (10.5%). Regarding multidrug resistance (MDR), PE harbored a higher proportion (84.2%) of MDR bacteria compared to 66.7% of the PP isolates. However, none of the differences observed between the polymer types reached statistical significance (*p* > 0.05, Chi-Square test) (Supplementary Table 1).

### 2.4 Prevalence of ESBL and carbapenemase-producing isolates

**FIGURE 3:**
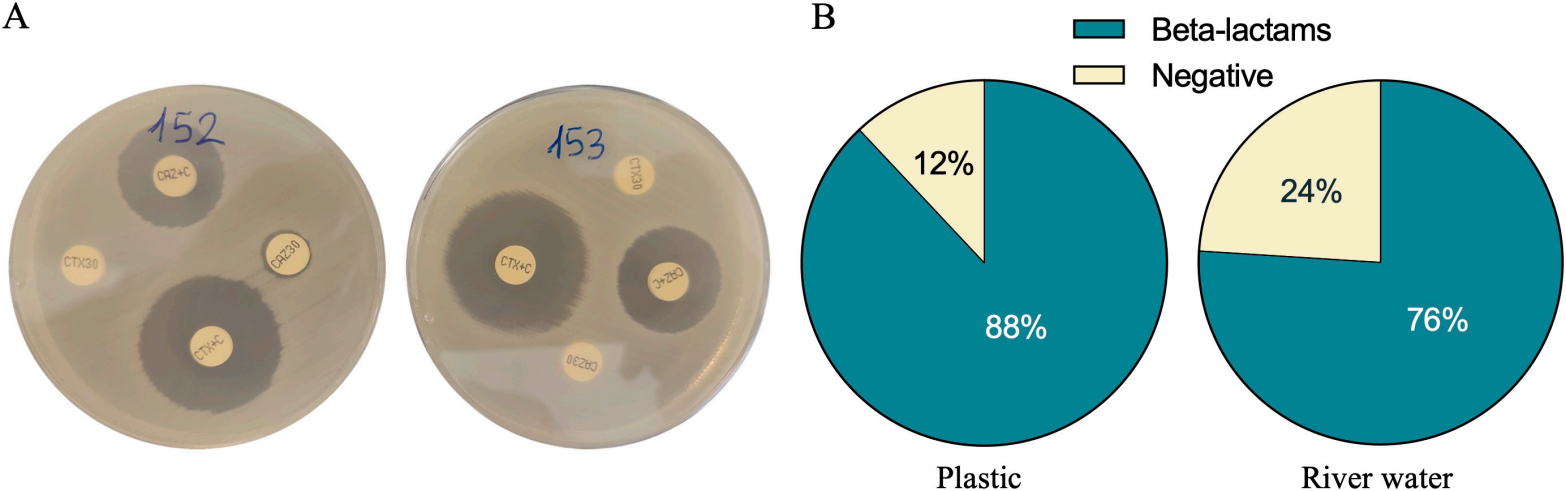
Phenotypic test to determine ESBL production. Two examples of 3GC-resistant *E. coli* isolates producing ESBLs **(A)**. Pie chart showing the distribution of ESBLs producers for both samples, plastic and water. Eighty-eight percent of the resistant strains from plastic and 76% from river water are ESBL producers **(B)**.

To further understand the resistance mechanisms to carbapenems of the environmental isolates, we investigated the production of extended-spectrum beta-lactamases (ESBLs) and carbapenemases, which represent two key resistance mechanisms in clinical isolates. The prevalence of extended-spectrum β-lactamase (ESBL)-producing isolates was high in both the polymer types (PE 95% and PP 81%). However, none of the differences observed between the polymer types reached statistical significance (*p* > 0.05, Chi-Square test) (Supplementary Table 1). Among the *Enterobacteriaceae* isolated from both plastic-associated and river water samples the prevalence of ESBL producer strains was 76 and 88%, respectively (Figures 3A,B).

Five of these strains (*C. freundii* 146, *K. pneumoniae* 175, *K. pneumoniae* 194, *E. cloacae* 236, *K. pneumoniae* 290) were KPC-producers, while two strains (*C. freundii* 167, *E. coli* 177) produced metallo-β-lactamases (MBLs). Out of the total isolates, 4% were plastic associated and 24% were river water carbapenemases producer strains. The presence of carbapenem resistance genes was investigated by molecular analysis. The *bla*_*KPC*_ gene was found in the strains: *C. freundii* 146, *K. pneumoniae* 175, *K. pneumoniae* 194, *E. cloacae* 236, *K. pneumoniae* 290; while the *bla*_*NDM*_ (New Delhi metallo-β-lactamase), was detected in the *C. freundii* 167 and *E. coli* 177 strains. Additionally, the *bla*_*VIM*_ gene (encoding the Verona integron–encoded metallo-β-lactamase) was identified in *C. freundii* 146 isolated from plastic associated biofilm.

### 2.5 Multi-locus sequence typing of KPC-producers *K. pneumoniae* and beta lactamase producers *E. coli strains*

Multi-Locus Sequence Typing (MLST) analysis was conducted to determine the genetic association between the three meropenem-resistant and KPC-producers *K. pneumoniae* isolates (175, 194, and 290) and the currently circulating lineages. These three strains exhibited a similar MDR pattern, including resistance to CTX, CAZ, LEV, CN, MEM, AMC, and FOX, and tested positive for *bla*_*TEM*_, *bla*_*SHV*_, and *bla*_*KPC*_ genes (Figures 2, 3). The analysis revealed that all the three isolates shared the same allelic profile: *gapA*: 54, *infB*: 3, *mdh*: 1, *pgi*: 1, *phoE*: 1, *rpoB*: 9, *tonB*: 79, which corresponds to the sequence type ST1519.

Similarly, this analysis was also performed on the four 3GC-resistant *E. coli* beta lactamase producers isolated from plastic (*E. coli* 322, 321, 328, and 340). The results revealed that *E. coli* 322 and 321 shared the same allelic profile: *dinB:* 6, *icdA:* 6, *pabB:* 4, *polB*: 2, *putP* 154, *trpA: F*7, *trpB:* 2, *and uidA:* 4, corresponding to the sequence type ST471. The remaining two isolates, *E. coli* 328 and 340, shared the same allelic profile: *dinB:* 7, *icdA:* 33, *pabB:* 18, *polB*: 2, *putP* 5, trpA: 8, trpB: 2, and *uidA:* 2, corresponding to the ST87.

### 2.6 Molecular evaluation of antibiotic resistance markers

Molecular analysis using PCR was performed to identify ARGs in bacterial strains previously analyzed phenotypically. Taking into account the absence of a statistical significance in the distribution of the antibiotic susceptibility rates in the isolates recovered from two plastic polymers, it was decided to carry out the test on 25 isolates randomly selected from both plastic types and 25 from river water.

**FIGURE 4:**
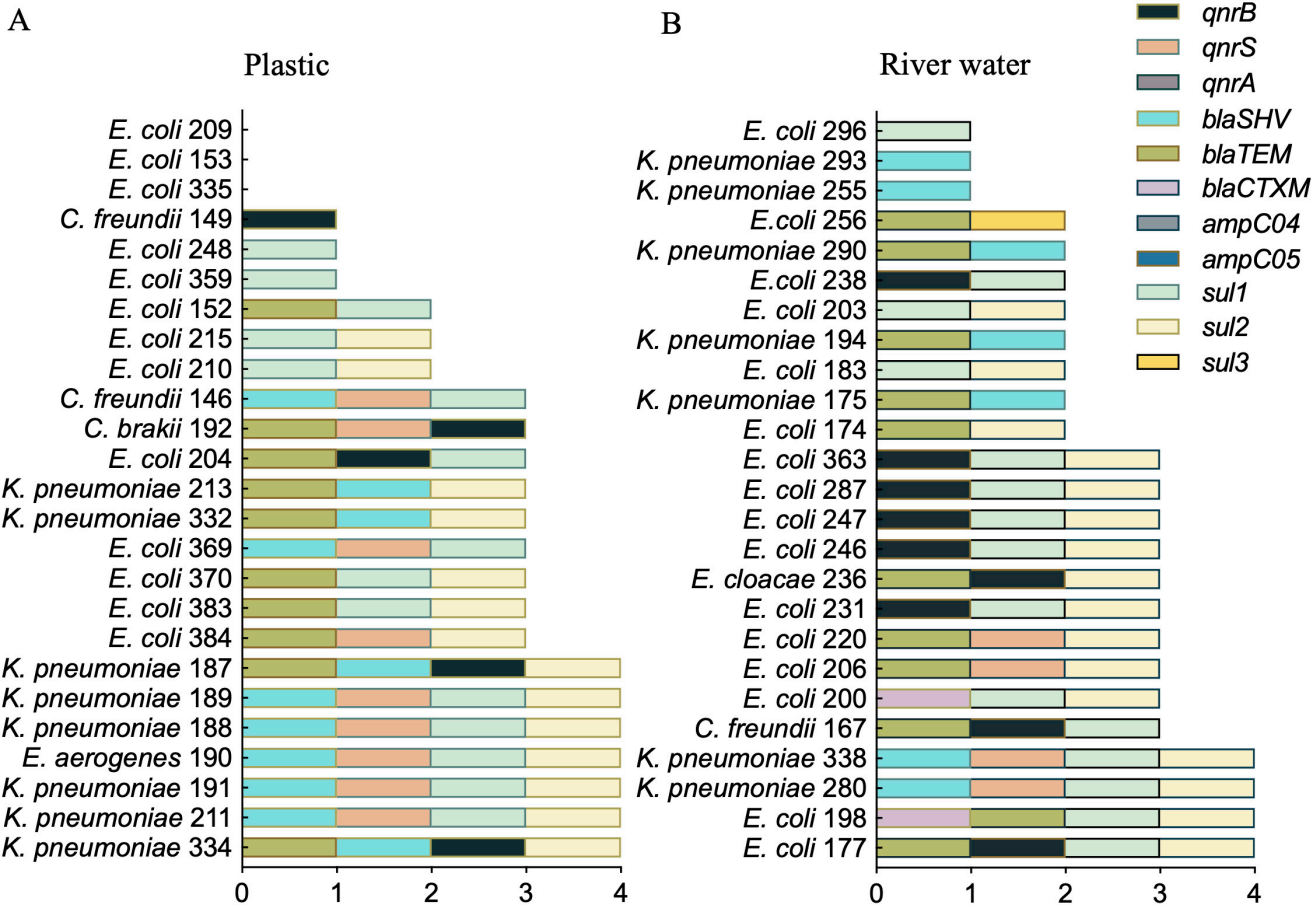
ARGs prevalence. Molecular detection by PCR of 11 ARGs in the bacterial strains isolated from plastic **(A)** or river water **(B)**.

All targeted ARGs were found in at least one isolate, except for *ampC04, ampC05* and *qnrA*. Comparing the AST results of the isolates with the ARGs detected, we observed that: (1) *sul* genes were consistently found in 100% (39/39) of trimethoprim/sulfamethoxazole (SXT) resistant strains; (2) the *qnr* genes (*qnrA*, *qnrB* and *qnrS*) were found in 40.6% (13/32) of levofloxacin (LEV) resistant strains; and at least one of the *bla* genes was found in 78% (32/41) of ESBL-positive strains (Figures 4A,B).

**FIGURE 5:**
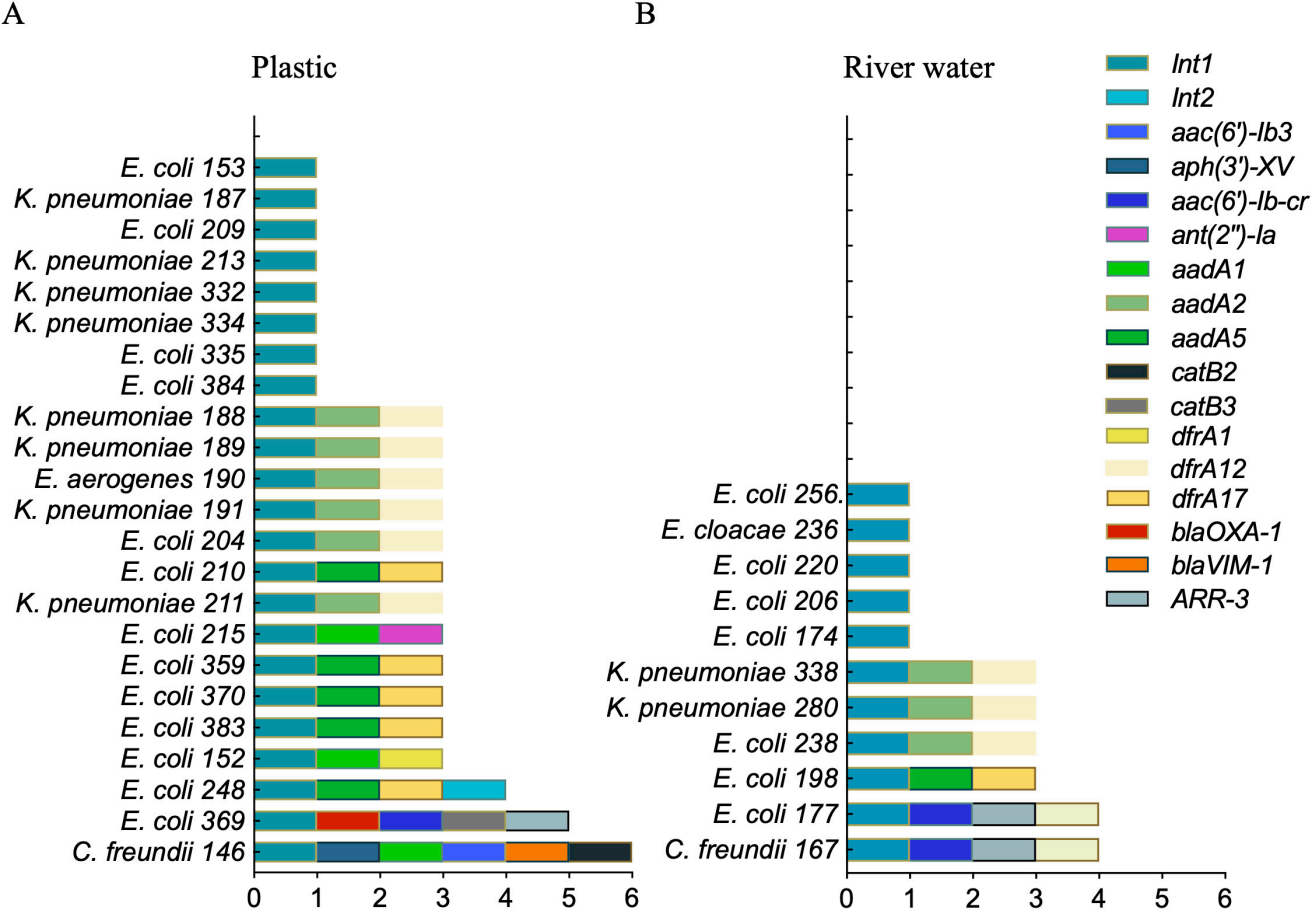
Diversity of the Class 1 Integron gene cassettes. Class 1 Integron gene cassettes in different bacterial species from plastic **(A)** and river water **(B)**. Seven gene cassettes arrays (combinations of colored boxes) have been identified. The presence of *Int1* was assessed by PCR, while ARGs associated to the gene cassettes were identified by Nanopore sequencing and analysis using the Integrall database.

In addition, we used PCR to detect the *Int1* gene as part of Class 1 Integron. The *Int1* gene was detected in 92% (23/25) of the isolates from plastics, with the 65% (15/23) of them harboring an associated gene cassette (Figure 5A). In contrast, only 44% (11/25) of the isolates from river water carried the *int1* gene, with the 55% (6/11) of these isolates containing an associated gene cassette carrying ARGs (Figure 5B). These data were confirmed using Nanopore sequencing and subsequent analysis by the Integrall database (Moura et al., 2009). Seven different cassette arrays were identified, containing ARGs associated to aminoglycosides [*aadA2, aadA5* and *ant(2”)-Ia*], trimethoprim (*dfrA17, dfrA12*, and *dfrA1*), chloramphenicol (*catB3*), and rifampicin (*ARR-3*) (Figure 5). Additionally, four *E. coli* isolates (183, 200, 203, and 296) missing the Class 1 integrase gene, contained a gene cassette composed of *aadA5* and *dfrA17* (data not shown). Overall, we observed a consistent presence of aminoglycoside resistance genes [*aadA2, aadA5*, and *ant(2”)-Ia*] within the gene cassettes. *E. coli* 369 contained *bla_*OXA*–1_* gene, encoding an oxacillinase. Similarly, *C. freundii* 146, carried the *bla_*VIM*–1_* gene encoding a metallo-beta-lactamase, according to both the AST and the molecular analysis. Furthermore, we identified a gene cassette containing Class 2 integrase (*Int2*) in *E. coli* 248 and *E. coli* 296. The identified *Int2* genes consist of a fragment that covers approximately 50% of the full sequence, resulting in a non-functional product. Among the most frequently detected gene cassette arrays, we observed a high prevalence of ARGs associated to aminoglycosides and trimethoprim resistances (represented in Figures 5A,B in green and yellow). Notably, 12 isolates from plastic exhibited this cassette array, compared to the 4 detected within isolates from river water. Overall, the gene cassette arrays of Class 1 Integrons detected in *Enterobacteriaceae* from plastic samples showed a higher heterogeneity in inserted resistance genes than those from water samples.

### 2.7 Plastisphere metagenomic analysis

**FIGURE 6:**
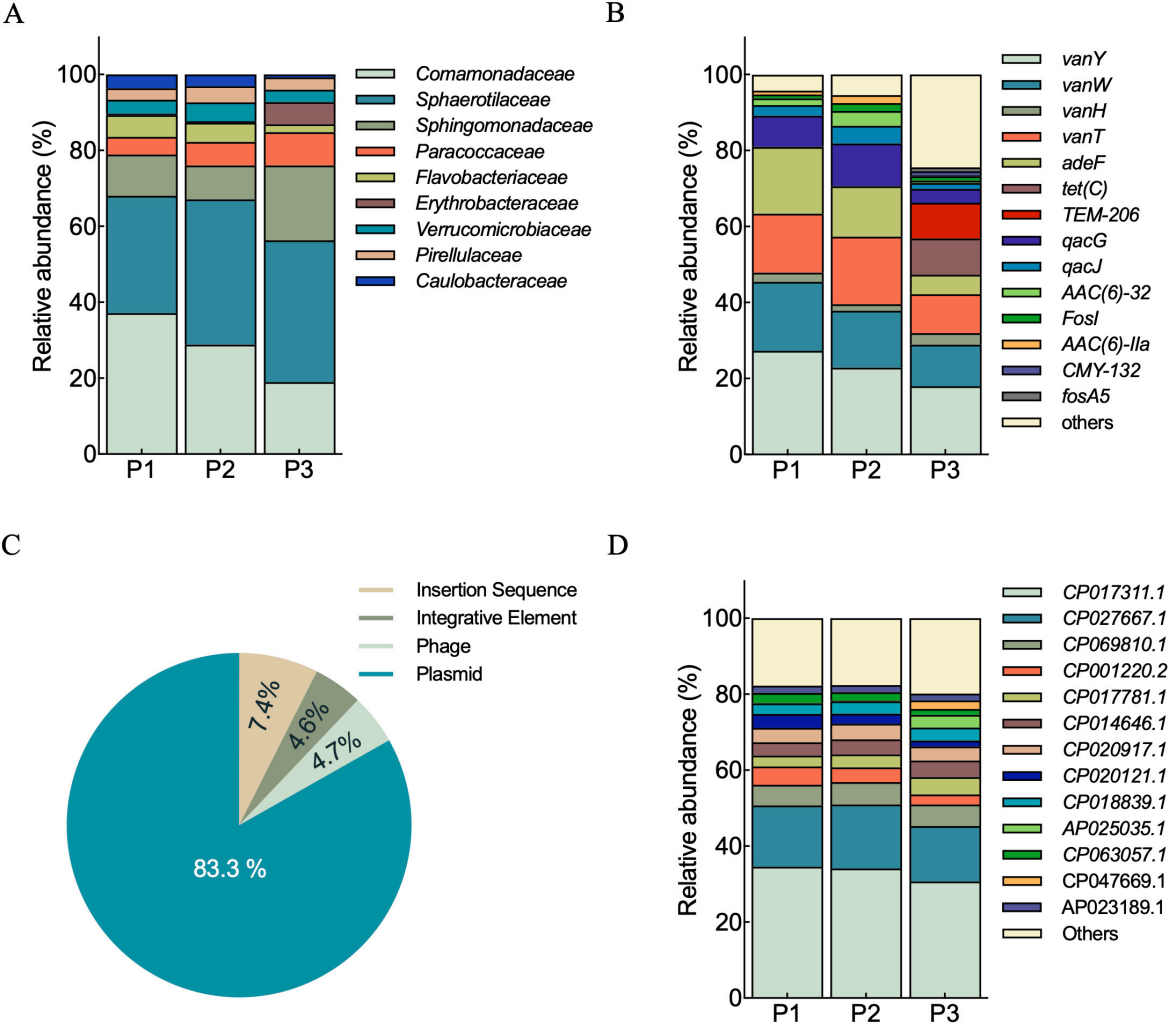
Plastisphere resistome. Metagenomic analysis based on shotgun sequencing data from P1, P2, and P3 plastisphere samples. Relative abundance of predominant bacteria at family level **(A)**. Distribution of antibiotic resistance genes (ARGs) identified from contigs (> 500 bp) in each sample site **(B)**. Relative abundance of mobile genetic elements (MGEs) across the three plastic samples **(C)**. Integrons identified from the Integrall Database (> 500 bp) in each sample **(D)**.

Shotgun metagenomic analysis of the plastisphere was used to study the microbial composition associated to plastic. A core microbiome of 1,442,182 genes was shared among the three plastic samples analyzed (Supplementary Figure 2). The taxonomic composition of the samples was dominated by bacteria, which accounted for approximately 75–80% of the microbial community in all samples. Despite the predominance of bacterial species, archaea, viruses, and eukaryotes were also present, but in smaller proportion. The taxonomic analysis of bacteria revealed that the biofilm attached to plastics is primarily composed of families commonly found in natural habitats. Among the dominant families, *Comamonadaceae*, *Sphaerotilaceae*, and *Flavobacteriaceae*, were consistently detected with variations in their relative abundance (Figure 6A). Less dominant taxa and those unclassified in databases, accounted for 64–67% of the total family-level microbial composition (Supplementary Figure 3).

At contig or unigene level, ARGs conferring resistance to clinically relevant antibiotics, such as vancomycin (*vanY*, *vanW*), tetracyclines (*tetC*), and beta-lactams (*bla_*TEM*–206_, bla_*CMY*–132_*), were detected in all sampling sites, with P3 exhibiting the highest diversity (Figure 6B). Additionally, other notable ARGs were identified, such as *adeF*, conferring resistance to fluoroquinolone and tetracycline antibiotics, *AAC(6’)*-*32* and *AAC(6’)*-*IIa*, associated with aminoglycoside resistance, and *FosI* and *FosA5*, which provide resistance to phosphonic antibiotics. Beyond antibiotic resistance genes, RGI identified the presence of genes involved in resistance to disinfectants, known to be involved in the co-selection mechanisms that promote the persistence and spread of ARGs (Basiry et al., 2022). Specifically, genes *qacG* and *qacJ* coding for resistance to quaternary ammonium compounds were identified in this study. Contig based analysis detected *Enterobacteriaceae* as carriers of multiple ARGs. Among the most relevant genes, *bla*_*TEM*_ conferring resistance to beta-lactams, and *tetA* linked to tetracycline resistance, were both identified within the same contig (*megahit_1986173*), suggesting their presence on the same genetic element. In addition to *bla*_*TEM*_ and *tetA*, other resistance determinants associated with *Enterobacteriaceae* were *hns, soxS, marA, mdtI*, and *csrA*, which are involved in regulatory pathways influencing antibiotic resistance. At genus level, *Klebsiella* also showed multiple ARGs co-localized within the same contigs. Among them, *oqxA* and *oqxB*, encoding resistance to fluoroquinolones, were found within *Klebsiella aerogenes* contigs (Supplementary Table 2). Short reads analysis revealed the presence of additional ARGs with high identity matches (> 85%) to the CARD database. This analysis identified genes such as *sul1, bla_*OXA*–20_, CRP, bla_*OXA*–10_, rsmA*, and others (Supplementary Figure 4). Among the identified *Enterobacteriaceae* by short reads analysis, we consistently found the presence of the *K. pneumoniae, E. coli* and *S. enterica* species. Additionally, we detected other species classified as priority pathogens by the WHO, including *E. faecium* (*Enterococcaceae*), *S. aureus* (*Staphylococcaceae*), *A. baumannii* (*Moraxellaceae*), *S. pneumoniae* (*Streptococcaceae*), and *M. tuberculosis* (*Mycobacteriaceae*).

The classification of MGEs across the three samples revealed that plasmids were the most prevalent, accounting for 83.3% of the total MGEs detected, followed by insertion sequences (7.4%), phages (4.7%), and integrative elements (4.6%) (Figure 6C). Conjugative elements represented the less abundant category accounting for the 0.02%. Integrons identified in the plastisphere were linked to specific bacterial hosts, based on accession numbers deposited in the Integrall database. All the identified integrons belong to the Class 1, except for the *CP001220.2* classified as the Class 2 Integron. Class 1 Integron integrase genes were detected in *Hydrogenophaga* sp. (CP017311.1) *Melaminivora* sp. (CP017311.1), *Comamonas testosteroni* (CP001220.2), *Rhodobacter* sp. (CP017781.1), *Thauera humireducens* (CP014646.1), and among bacteria of clinical importance like *Citrobacter freundii* (AP025035.1), *Klebsiella aerogenes* (CP047669.1) and *Pseudomonas* sp. *TUM18999* (AP023189.1), commonly harboring multidrug-resistant traits (Figure 6D).

## 3 Discussion

Plastic pollution is rising due to the steadily increase of global production. This phenomenon has a considerable impact in freshwater systems, which act as conduits for transferring pollutants from land to oceans (Dauvergne, 2018; Law, 2017). Concurrently, AMR is recognized as a growing threat that extends far beyond clinical settings and require a One Health approach. A relevant problem in this regard is the worldwide spread, in the last decades, of *Enterobacteriaceae* resistant to third-generation cephalosporins. Very few therapeutical treatments are currently available when this resistance is combined with the resistance to other first line agents, such as fluoroquinolones or aminoglycosides (Rohde et al., 2018). Our study highlights the intersection of these two global threats, through a multidisciplinary approach involving the development of an experimental setup mimicking plastisphere formation in a riverine environment for: (i) the evaluation of biofilm grown on plastics, (ii) the characterization of critical multidrug-resistant (MDR) bacteria and their antibiotic resistance genes (ARGs), and (iii) a comprehensive metagenomic analysis of the microbial communities associated to plastics.

A high number of 3GC-resistant *E. coli* and coliforms were isolated from plastics and river water, suggesting that both sources support the survival/proliferation of 3GC-resistant *Enterobacteriaceae*. Most of these critical isolates, belonging to species previously detected in riverine environment (Delgado-Blas et al., 2021; Piccirilli et al., 2019), display a MDR profile that represent a public health risk (Figure 2). Notably, a high prevalence of ESBL-producing bacteria was also observed (Figure 3), reinforcing the need for an integrated environmental surveillance on ESBLs producers (emphasized by the WHO Tricycle protocol), to better understand the pathways of the AMR spread (World Health Organization [WHO], 2021). The detection of at least one s*ul* gene in 80% of the isolates, indicates the widespread sulfonamide resistance and its environmental distribution, suggesting a strong selective pressure by sulfonamides on bacterial population inhabiting the river ecosystem.

The evidence that the plastisphere represents a hotspot for the acquisition and spread of ARGs (Stevenson et al., 2024) is supported by the high prevalence of Class 1 Integrons. This genetic element showed greater heterogeneity and length of associated gene cassette in isolates from plastics compared to those from river water, aligning with previous studies of bacteria associated to microplastic in aquatic environment (Zhang et al., 2020; Figure 5). These gene cassettes encode a diverse array of resistance determinants that can be horizontally transferred across different species, enhancing the adaptive potential of bacterial populations (Stalder et al., 2012). The presence of integrons in both environmental and clinical bacteria may also indicate their role as mediators of genetic exchange and their ability to mobilize ARGs between non-pathogenic and pathogenic bacteria, enhancing the overall adaptability of microbial communities (Bhat et al., 2023).

In our study, the plastisphere core microbiome was dominated by the *Comamonadaceae* family, in accordance with studies performed on plastic substrates (Donnarumma et al., 2024). Although 3GC-resistant *Enterobacteriaceae* were not detected through shotgun metagenomic analysis, probably due to their low abundance, these results show the importance of integrating complementary approaches to comprehensively understand the diversity and complexity of the plastisphere community.

Among ARGs identified by contig-based analysis, the most abundant resistance determinants were *bla_*TEM*–206_*, *bla_*CMY*–132_* (beta-lactams), *tetC* (tetracyclines), and *vanY*, *vanW* (vancomycin). These data confirmed that river plastisphere may harbor various ARGs, especially those conferring resistance to clinically important antibiotics such as, glycopeptides and β-lactams (Guruge et al., 2024). Additionally, the concomitant detection of disinfectant resistance genes *qacJ* e *qacG*, further highlights the plastisphere’s potential to adapt to and to spread resistance beyond antibiotics. In environments exposed to anthropogenic activities (such as agriculture runoff or farming practices) disinfectants are known to exert selective pressure, not only promoting the persistence of resistant bacteria but also increasing the mutation rate and enhancing their ability to acquire antibiotic resistance (Randall et al., 2004). Short-read analysis revealed the presence of additional ARGs, including *sul1, bla_*OXA*–20_*, and *bla_*OXA*–10_*, which were not detected in contig-based assemblies. This finding suggests the presence of a broad and complex resistome associated with plastics, where low-abundance or fragmented genetic elements may escape conventional assembly approaches. The presence of MGEs, among which plasmids were the most abundant, support the hypothesis that plastics may act as an enhancer of HGT by promoting bacterial proximity and providing specific micro-habitat favoring the gene transfer (Ahmad et al., 2024; Ferheen et al., 2025). Overall, these results demonstrated the importance of a complementary and multidisciplinary study combining culture-dependent and metagenomic analyses to comprehensively reveal the resistome of plastic-associated microbial communities.

Among 3GC-resistant *Enterobacteriaceae*, 7 CRE were detected, further reinforcing the public health risks posed by freshwater in the spread of critical priority pathogens that potentially may enter or re-enter humans by commensals pathways. Notably, three of the CRE isolates, specifically KPC-producing *Klebsiella pneumoniae*, were identified as ST1519, a sequence type previously reported, in Italy, only in hospitals, highlighting the tight connection between clinical and environmental settings. This evidence may reflect inputs from anthropogenic sources, such as municipal wastewater, hospital effluents, or human activities that can introduce clinically derived bacteria into the river. This is consistent with the sampling sites, characterized by industrial and agricultural activities, and for which have been reported chemical and microbial pollution (Bacino Chienti, 2018–2020). Such findings raise questions about the mechanisms enabling high-priority pathogens to persist in natural ecosystems and potentially move among environments, animals, and humans (Ding et al., 2023; Larsson and Flach, 2022). More efforts are required to monitor the dynamics of these critical priority bacteria in river ecosystems. Only few studies reported the presence of CRE in Italian rivers (Piccirilli et al., 2019; Rimoldi et al., 2021) and across Europe in the Lis River (Portugal), the Danube River, and in surface waters in Switzerland (Teixeira et al., 2022; Kittinger et al., 2016; Bleichenbacher et al., 2020).

In conclusion, our results demonstrate that the plastisphere may act as a genetic reservoir for antibiotic resistances, amplifying the risk of ARGs being transferred to clinically significant pathogens. This evidence is further supported by a recent study showing that riverine plastisphere contribute to the accumulation and transport of antibiotic-resistant pathogens and clinically relevant ARGs (Silva et al., 2023). However, the relatively short exposure of plastic in this study, does not allow the evaluation of seasonal or long-term variations in biofilm composition or resistome changes. Further research is needed to elucidate mechanisms involved in ARGs mobilization within rivers impacted by pollution. Such evidence will be fundamental to restrain the spread of AMR from environmental reservoirs to clinical settings, as well as to prevent the transfer of clinically derived resistances from hospitals into natural environments.

## 4 Materials and methods

### 4.1 Study area and sampling sites

The area of the river investigated in this study is in the Marche Region (central Italy), where the presence of antibiotic-resistant pathogens and their ability to colonize plastic fragments has never been investigated before. In particular, the Chienti River was chosen as one of the main rivers in this region that presents a gradient of land use and anthropogenic pressures along its course. Three different geographical locations along the course of the Chienti river were selected as sampling sites, namely Pontelatrave (PLT), Corridonia (CORR) and Montecosaro (MONT). These sites have been selected based on data emerged from the last report on the monitoring of Chienti river (Bacino Chienti, 2018–2020) by Agenzia Regionale per la Protezione Ambientale delle Marche (ARPAM).

These sampling sites show the following main features:

1. The site of Pontelatrave (x: 2363793; y: 4771352; ARPAM monitoring station: R110195CH) is the closest to the source of the river (14 km). In this section the watercourse has moderate current velocity, with an average flow rate measured in 2021 of 1923 l/s. The use of the surrounding land is mainly agricultural, so the environmental pressure is represented by agricultural (crop production and livestock farming) use and rural transportation. In this site ARPAM reported the presence of pesticides and microbial pollution by *E. coli*.
2. The site of Corridonia (x: 2399783; y: 4792022; ARPAM monitoring station: R1101913CH) is 40 km downstream from the Pontelatrave site along the river. The watercourse is affected by daily variation of the flow due to the release by the hydroelectric power stations located upstream. The surrounding area shows a higher population density compared to PLT and the land use is for agriculture, horticulture and industry (small and medium enterprises). Different environmental pressures were reported: chemical pollution (pesticides, phthalates, metals), urban runoff, agriculture and transport.
3. Montecosaro (x: 2409404; y: 4791483; ARPAM monitoring station: R1101914CH) is located 7 km upstream the river mouth and in this section the current velocity is moderate, with limited turbulence. Similarly to CORR, the surrounding area shows a higher population density compared to PLT and its land use is for agriculture, horticulture and industry. Environmental pressures were reported: chemical pollution (pesticides, metals), nitrogen pollution, urban runoff, agriculture use and transport (Bacino Chienti, 2018–2020).

### 4.2 Experimental design and set-up

**FIGURE 7:**
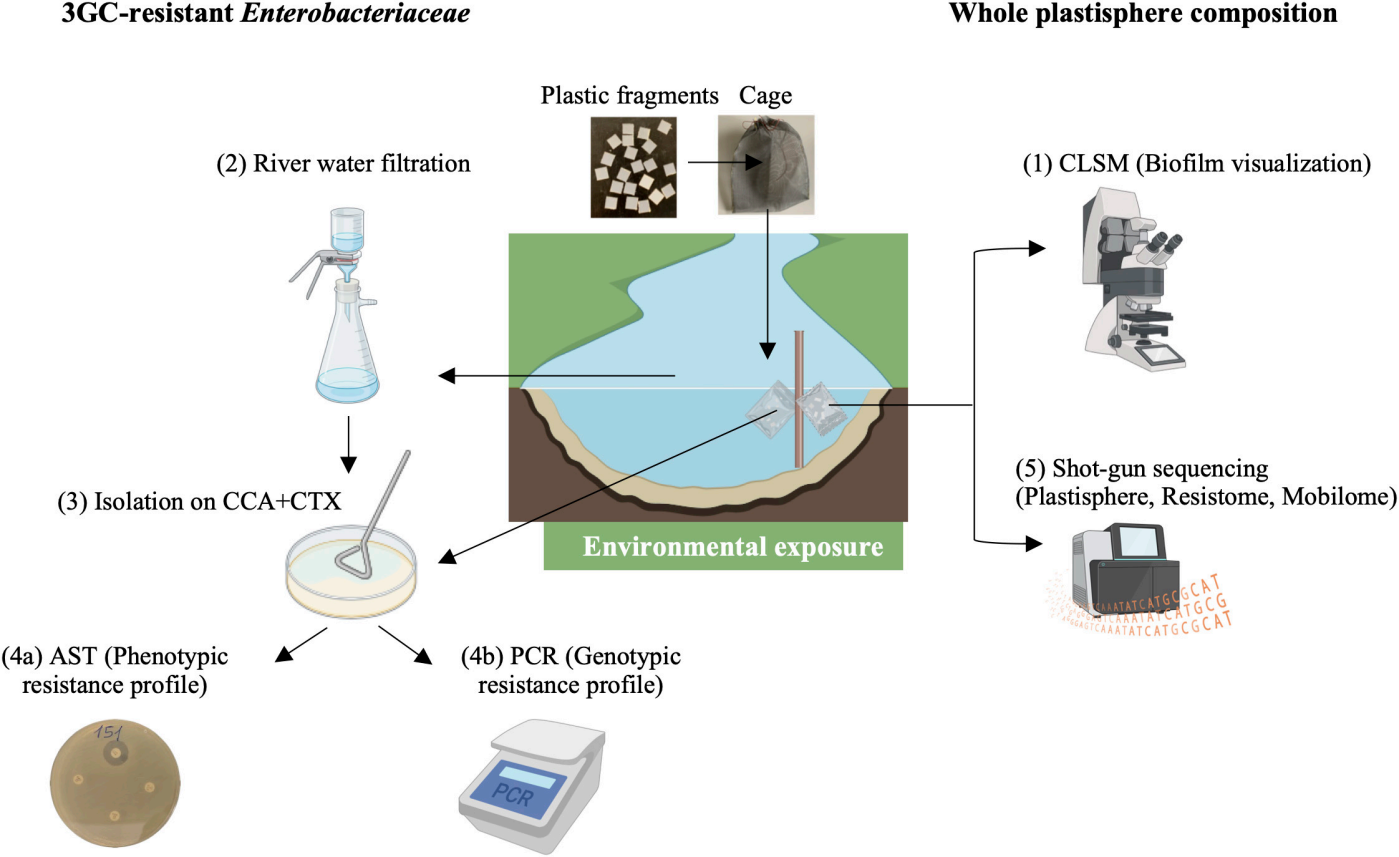
Overview of the study. Plastic fragments were inserted into filter cages and submerged in the river in three distinct sites of the Chienti River for 3 weeks. River water and plastic fragments were collected and analyzed weekly. Workflow of the study included: (1) Confocal Laser Scanning Microscopy analysis to detect biofilm associated to plastic fragments; (2) River water sampling and filtering by 0.45 μm (3) Bacterial isolation from river water and plastic samples on CCA medium with 8 mg/L Cefotaxime and subsequent biochemical identification; (4a) Antimicrobial susceptibility testing (AST); (4b) Molecular analysis to identify ARGs and Class 1 integron gene cassettes; (5) Metagenomic analysis to characterize the microbial community of the plastisphere, and its associated ARGs and MGEs.

A multi-step protocol was established with the aim of identifying 3GC-resistant *Enterobacteriaceae* isolated from both plastic fragments and river water (Figure 7). At first, we deliberately introduced caged plastic fragments (1 × 1 cm) into three different sites along the Chienti river (Marche, Italy), allowing biofilm formation over 3 weeks (Reis and Aldridge, 2024). Two hundred plastic fragments of PE and PP (purchased from Merck™), selected due to their widely use in daily plastic product, were inserted in nylon net bags that were firmly anchored to the side banks through a steel wire (Rodrigues et al., 2019). The 3GC-resistant *Enterobacteriaceae* were further studied using microbiological and molecular techniques. Sixty-five 3GC-resistant *Enterobacteriaceae* were isolated and biochemically identified. Their phenotypic antimicrobial resistant profile has been characterized through the disk diffusion susceptibility test, according to EUCAST guidelines, targeting the classes of sulfonamides, quinolones, beta-lactams and carbapenems. The presence of Class 1 Integron was detected by PCR, and their associated gene cassettes were sequenced to identify their associated ARGs. As a last step, shotgun metagenomic analysis was performed on the total DNA extracted from plastic associated biofilms to characterize the microbial community, resistome, and MGEs. This approach allowed us to characterize the microbial community associated to the plastisphere, including uncultivable bacteria, complementing the data provided by microbiological and molecular assays.

### 4.3 CLSM analysis

Plastic fragments (25 PP and 25 PE) and water samples (1 L) were collected weekly from each site for 3 weeks. The samples were immediately refrigerated at + 4°C, transported to the laboratory and analyzed within 24 h. Three fragments of PP and PE were stained with SYTO9 dye (LIVE/DEAD^®^ BacLight Bacterial Viability Kit-Thermofisher™). Each plastic fragment was submerged in sterile saline containing SYTO9 dye (at a final concentration of 0.5 μM) and incubated at room temperature in the dark for 15 min. Subsequently, fragments were mounted between a slide and a coverslip and observed using a 100X objective under a C2 + Laser Scanning Confocal Microscope (Nikon, Tokyo, Japan).

### 4.4 Isolation of 3GC-resistant *Enterobacteriaceae* and biochemical identification

Twelve fragments of each polymer, PP and PE, from each sampling site were mild sonicated using a microtip in 15 ml of sterile saline (9 g/L NaCl) solution under the following conditions: 4 pulses of 45 s each at 9 W, separated by a 1-min interval. The post-sonicated solutions (100 μL, 1 mL, 10 mL each) in parallel with river water (10 and 100 mL each) were cultured to isolate cefotaxime-resistant coliforms and *E. coli* by the membrane filtration method. The nitrocellulose membranes (0.45 μm Millipore) were transferred into Coliform Chromogenic Agar medium (CCA) supplemented with cefotaxime (8 μg/mL). Plates were incubated at 37°C for 24 h, then colonies were counted and isolated. Cefotaxime-resistant strains were stored in 20% glycerol stocks at –80°C for the subsequent analysis. Isolates resistant to cefotaxime were subjected to oxidase test (Oxoid™). Oxidase negative isolates were biochemically identified using API 20 E™ strips in combination with APIWEB software (Biomerieux™). Overnight bacterial suspension was diluted to a turbidity of McFarland 0.5 in saline. The suspension was inoculated in the micro-tubes of the API 20E strip and then incubated at 37°C for 18–24 h.

### 4.5 Antibiotic susceptibility testing

The antibiotic susceptibility profiles of cefotaxime-resistant *Enterobacteriaceae* strains were determined using the disk diffusion method.^1^ The antibiotics tested were cefotaxime (CTX, 5 μg), ceftazidime (CAZ, 10 μg), gentamicin (CN, 10 μg), levofloxacin (LEV, 5 μg), cefoxitin (FOX, 30 μg), trimethoprim/sulfamethoxazole (SXT, 1.25/23.75), meropenem (MEM, 10 μg), amoxicillin/clavulanic acid (AMC, 20/10 μg), and tigecycline (TGC, 15 μg). After incubation at 37°C for 18 ± 2 h, the diameter of inhibition zones was measured and interpreted according to EUCAST guidelines. ESBL Confirm Kit (ROSCO Diagnostica™, Denmark) was used to detect Extended-Spectrum Beta-Lactamases (ESBLs). The kit includes antibiotic tablets (Cefotaxime 30 μg, Ceftazidime 30 μg and Cefepime 30 μg) and their combination with Clavulanate, an inhibitor of ESBLs. After the incubation at 35 ± 1°C for 18 ± 2 h the diameter of the inhibition zones was measured. If the inhibition zone diameter around the combination disc is ≥ 5 mm larger than the one around the single cephalosporin disc/tablet the isolate is showing ESBL production. Similarly, the Meropenem-resistant strains were tested using the KPC/MBL and OXA-48 Confirm Kit- (ROSCO Diagnostica™, Denmark). The interpretation of phenotypic mechanisms of resistance to carbapenems was obtained according to the kit user manual.

### 4.6 DNA extraction

The genomic DNA of the isolated cefotaxime-resistant *Enterobacteriaceae* strains were extracted using the GenElute™ DNA Kit (Merck). Bacterial cultures were grown overnight in LB media at 37°C, and DNA extraction was performed according to the manufacturer’s instructions. Total bacterial communities associated to plastic samples, collected from Pontelatrave (P1), Corridonia (P2) and Montecosaro (P3) were detached from 90 plastic fragments per sample (mixing PE and PP) using a mild sonication protocol (see section 4.3). The total DNA was extracted using EZNA water DNA kit (Omega Bio-Tek™) and assessed for quality through gel electrophoresis and Nanodrop spectrophotometry.

### 4.7 DNA amplification and sequencing

PCR amplification of ARGs *qnrB, qnrS, qnrA* (quinolones)*, bla_*SHV*_, bla_*TEM*_, bla_*CTX*–*M*_, ampC04, ampC05* (beta-lactamases)*, sul1, sul2*, and *sul3* (sulfonamides) and *Int1* gene (Table 1; Zhang et al., 2020) was performed using TaKarRa Taq™ DNA Polymerase. The reaction mixture (25 μl) contained: Takara Taq™ DNA Polymerase 0.63 units, 1X PCR Buffer, 200 μM dNTPs, 2 μL of template gDNA, 0.5 μM each of forward and reverse primers. The amplification conditions were the following: initial denaturation at 94°C for 5 min, followed by 30 cycles of denaturation at 94°C for 30 s, annealing according to Table 1 for 30 s, and extension at 72°C for 30 s. A final extension step was carried out at 72°C for 1 min. The variable region of the Class 1 Integron was amplified using primers targeting the conserved regions flanking this segment (Zhang et al., 2020). For Class 1 gene cassette amplification (Table 1) the Jump Start Accu Taq™ DNA Polymerase was used. The reaction mixture (25 μL) contained Jump Start Accu Taq™ DNA Polymerase at a concentration of 1.25 units, 1X PCR Buffer, 200 μM dNTPs, 2 μL of genomic DNA, 0.5 μM forward and reverse primers each. The thermal cycler conditions were as follows: 5 min at 94°C, followed by 30 cycles of 30 s at 94°C, 30 s at 55°C for annealing, and 5 min at 72°C for extension. A final extension step at 72°C for 10 min was included. PCR products were then analyzed by agarose gel electrophoresis. The Class 1 gene cassette PCR products were purified using the GenElute™ PCR Clean-up Kit (Merck) and quantified by Nanodrop spectrophotometer. Purified DNA was sequenced at Plasmidsaurus (United States), by Nanopore Sequencing.

**TABLE 1:** Oligonucleotides targeting ARGs and *Int1* genes.

Gene	Orientation	Sequence*	Amplicon size (bp)	Annealing t (°C)
*sul1*	FW	CGCACCGGAAAACATCGCTGCAC	162	67
	RV	TGAAGTTCCGCCGCAAGGCTCG		
*sul2*	FW	TCCGGTGGAGGCCGGTATCTGG	190	65
	RV	TTCGTTCACGCCTTACCCAGC		
*sul3*	FW	TCCGTTCAGCGAATTGGTGCAG	127	63
	RV	TTCGTTCACGCCTTACACCAGC		
*qnrA*	FW	AGGATTTCTCACGCCAGGATT	124	59
	RV	CCGCTTTCAATGAAACTGCA		
*qnrB*	FW	CAGATTTYCGCGGCGCAAG	134	55
	RV	TTCCCACAGCTCRCAYTTTTC		
*qnrS*	FW	GTATAGAGTTCCGTGCGTGTGA	189	60
	RV	GGTTCGTTCCTATCCAGGATT		
*ampC*	FW	TCCGGTGACGCGACAGA	64	60
	RV	CAGCACGCCGGTGAAAGT		
*bla_*CTX*–*M*_*	FW	GGAGGCGTAGACGGCTTTT	53	60
	RV	TTCAGTGCGATCCAGACGAA		
*bla*_*SHV*_	FW	TCCATGATGGCACCTTTAAA	90	58
	RV	TTCGTCACCGGCATCCA		
*bla*_*TEM*_	FW	AGCATCTTACGGATGGCATGA	101	60
	RV	TCCTCCGATCGTTGTCAGAAGT		
*Int1*	FW	GGCTTCGTGATGCCTGCT	146	60
	RV	CATTCCTGGCCGTGGTTCT		
*Int1V*	FW	TCATGGCTTGTTATGACTGT	variable	55
	RV	GTAGGGCTTATTATGCACGC		

*Primer sequences used as reported by Zhang et al. (2020).

### 4.8 Multi locus sequence typing

The MLST typing analysis was conducted according to the scheme developed by Pasteur Institute (2025).^2^ The analysis on the three meropenem-resistant *K. pneumoniae* isolates is based on Sanger sequencing of seven PCR-amplified housekeeping genes: *gapA*, *infB*, *mdh*, *pgi*, *phoE*, *rpoB*, and *tonB*. Analysis on 3GC-resistant *E. coli* isolates was performed on eight housekeeping genes: *dinB*, *icdA*, *pabB*, *polB*, *putP*, *trpA*, *trpB* and *uidA.* Sequence types (STs) were assigned according to the allelic profiles of the isolates found on the BIGSdb-Pasteur platform.

### 4.9 Metagenomic analysis

Shotgun metagenomic analysis of the plastisphere was used to study the microbial composition associated to plastic as well as to further inquire on their AMR profiles. Three plastic samples, P1, P2, and P3, were submerged in river water and then collected at Pontelatrave, Corridonia and Montecosaro, respectively. Preparation of DNA libraries, sequencing and assembly of contigs have been performed by Novogene Europe (Germany), using Illumina sequencing technology. Bioinformatic analyses were performed on 30 Gbyte of raw sequence data per sample. The quality and the presence of adapters of the reads were evaluated using FastQC.^3^ Subsequently, adapter sequences and low-quality reads (Phred score < 30) were removed using Trim Galore!.^4^ After quality control, the clean data from each sample were used for metagenome assembly (> 500 bp) using MEGAHIT software. Gene prediction (structural annotation) was performed with MetaGeneMark. Unigene sequences were aligned and annotated using DIAMOND software^5^ (Buchfink et al., 2015) and Micro_NR database, which includes sequences from bacteria, fungi, archaea, and viruses extracted from NCBI’s NR database.^6^ Short reads were also analyzed for taxonomic classification using Kraken2. For antibiotic resistance genes detection at read and contig level, the CARD database (McArthur et al., 2013) was used with RGI (Resistance Gene Identifier) (Jia et al., 2017). Integrons and MGEs were identified by aligning contigs against Integrall database (Moura et al., 2009) and MobileOG database (Brown et al., 2022), respectively.

### 4.10 Statistical analysis

Significance of results was calculated in Python using Google Collab. Differences were evaluated using the *Chi-square* test, with a *p* < 0.05 considered statistically significant.

## Data Availability

The data presented in this study are deposited in the NCBI BioProject database under BioProject ID PRJNA1365058.

## Footnotes

1
www.eucast.org

2
https://bigsdb.pasteur.fr

3
www.bioinformatics.babraham.ac.uk/projects/fastqc/

4
https://www.bioinformatics.babraham.ac.uk/projects/trim_galore/

5
https://github.com/bbuchfink/diamond/

6
https://www.ncbi.nlm.nih.gov/
