# Supplementary material for: The plastisphere and river systems as reservoirs for antibiotic resistant bacteria
Source: Front Microbiol. 2026 Jan 22;16:1721325. doi: 10.3389/fmicb.2025.1721325 (PMC12875321; doi:10.3389/fmicb.2025.1721325)
Supplement: Supplementary file 1 [file Table_1.docx]

**Supplementary Materials**

**Supplementary Figure 1. Plastisphere visualization.**  Bacteria attached to PE and PP fragments (two sample each) after being exposed to river water, stained by Syto9 and observed by Confocal Laser Scanning Microscopy (CLSM). The square images show biofilm projection through the x-y plane. The scale bars are 10 μm.

**Supplementary Table 1. Percentage of Antibiotic Resistances of bacteria attached to PE and PP.**

|  | % | | | | | | | | | |  |
| --- | --- | --- | --- | --- | --- | --- | --- | --- | --- | --- | --- |
| Polymer | CTX | CAZ | CN | LEV | SXT | MEM | AMC | TGC | FOX | MDR | ESBLs |
| PE | 100 | 100 | 47 | 68 | 73.7 | 0 | 84.2 | 0 | 10.5 | 84.2 | 95 |
| PP | 100 | 100 | 14 | 19 | 85.7 | 4.8 | 85.7 | 0 | 28.6 | 66.7 | 81 |

* Chi-Square test was performed (p > 0.05), and no values were found to be statistically significant.

**Supplementary Figure 2.** **Core genome and composition of microbial communities associated to plastics.** Venn diagram genome analysis based on the abundance of genes in every sample.

The analysis identifies a core pan-genome of approx. 1,442,182 genes.

**Supplementary Figure 3. Plastisphere taxonomic composition.** Relative abundance of bacterial families identified from the contigs in each plastic sample (P1, P2, P3)

**Supplementary Figure 4. Short reads analysis on ARGs.** Percentage identity of ARG detected from short reads using the CARD database. Identity values ranged between 89.4 to 100%.

**Supplementary Table 2. ARGs -Associated Enterobacteria Identified Through Contig Analysis**

| **Gene name** | **Taxa** | **Contig ID** | **ORF ID** |
| --- | --- | --- | --- |
| *hns* | *Enterobacteriaceae* | megahit_372973 | megahit_372973_36616-37023 |
| *soxS* | *Enterobacteriaceae* | megahit_543111 | megahit_543111_2109-2438 |
| *marA* | *Enterobacteriaceae* | megahit_679370 | megahit_679370_2932-3315 |
| *mdtI* | *Enterobacteriaceae* | megahit_956635 | megahit_956635_6099-6428 |
| *csrA* | *Enterobacteriaceae* | megahit_1116438 | megahit_1116438_6185-6370 |
| *crp* | *Enterobacteriaceae* | megahit_1415717 | megahit_1415717_8293-8925 |
| *blaTEM* | *Enterobacteriaceae* | megahit_1986173 | megahit_1986173_1-816 |
| *tetA* | *Enterobacteriaceae* | megahit_1986173 | megahit_1986173_2833-4023 |
| *marR* | *Klebsiella* | megahit_679370 | megahit_679370_2480-2914 |
| *emrB* | *Klebsiella* | megahit_1206480 | megahit_1206480_6745-8283 |
| *fosA5* | *K. aerogenes* | megahit_22363 | megahit_22363_9045-9464 |
| *oqxA* | *K. aerogenes* | megahit_38292 | megahit_38292_857-2032 |
| *oqxB* | *K. aerogenes* | megahit_38292 | megahit_38292_2056-5208 |
| *ftsI* | *K. aerogenes* | megahit_359521 | megahit_359521_2759-4525 |
| *mdtJ* | *K. aerogenes* | megahit_956635 | megahit_956635_6415-6777 |
| *ddl* | *K. aerogenes* | megahit_1127827 | megahit_1127827_11421-12518 |
| *emrA* | *K. aerogenes* | megahit_1206480 | megahit_1206480_8299-9471 |
